# Masson's tumor (intravascular papillary endothelial hyperplasia)^☆^^☆☆^

**DOI:** 10.1016/j.abd.2019.09.013

**Published:** 2019-09-30

**Authors:** Sergio Emerson Sasso, Ana Paula Naspolini, Tassiana de Boit Milanez, Guilherme Suchard

**Affiliations:** a Department of Undergraduate Medical Studies, Universidade do Extremo Sul Catarinense, Criciúma, SC, Brazil; b Department of Anatomical Pathology, Laboratório Rocha, Criciúma, SC, Brazil; c Department of Ultrasonography, Unidade Radiológica Criciúma, Criciúma, SC, Brazil

Dear Editor,

In 1923, Masson initially described a process, at that time neoplastic, which consisted of vascular lumen obliteration due to papillary endothelial hyperplasia associated with degenerative changes.^1^ Because of its similarity to angiosarcoma, this entity was termed vegetative intravascular hemangioendothelioma, or simply Masson's tumor; denominations such as intravascular angiomatosis, Masson's pseudo-angiosarcoma, Masson's hemangioma, and Masson's vegetative intravascular hemangioendothelioma have also been used.^2^, ^3^, ^4^ A reactive and therefore non-neoplastic character of the disease is currently postulated. Consequently, since its reclassification by Clearkin and Enzinger in 1976, the denomination used is the intravascular papillary endothelial hyperplasia (IPEH). The exact etiology remains unclear and appears to be multifactorial. Secondary factors such as local trauma and previous vascular conditions (hemangiomas, vascular malformations, pyogenic granulomas, blood stasis, etc.) account for 30% of the cases, but in 70%, the triggering stimulus cannot be identified. Excessive endothelial proliferation stimulated by local production of growth factors has been a promising hypothesis to explain the etiology of IPEH.^3^ Studies using northern blot and immunoblotting revealed a significant increase in the expression of fibroblast growth factor beta (FGF-β), suggesting its secretion by the endothelial cells through an autocrine mechanism.^4^ IPEH is a rare vascular disorder, accounting for about 2% of all tumors. The lesion is usually found in soft tissues from areas exposed to trauma. It can affect any part of the body, but the places of greatest concern are the head, neck, and upper extremities. It is slightly more common in women (1.3:1) and there is no preference for age. Clinically, IPEH presents as firm or softened surface nodules, not adhered to the superficial and/or deep planes. It presents blue-red coloration (resulting from dilation and obstruction of the endovascular space) and does not present pulsatility.^2^, ^5^ It may eventually produce clinical features that mimic a neoplasia, justifying the need for its recognition by the physician.^1^ We report the case of an 11-year-old female patient who presented an asymptomatic nodular lesion in the left upper limb with about three months of evolution. She reported rapid growth of the lesion and denied a history of local trauma. At the dermatological examination we noticed the presence of a subcutaneous nodule with a reddish-blue color, poorly delimited, with fibroelastic consistency, not adhered to superficial or deep planes, non-pulsatile and painless to palpation, measuring about 1.5 cm in diameter, and located in the third of the extensor region of the left forearm above the ulnar diaphysis (Fig. 1). A soft, hypoechogenic subcutaneous nodule with lobulated contours was observed, measuring 1.4 × 1.1 × 0.6 cm, and Doppler ultrasonography showed it to be intensely vascularized (Fig. 2). An excisional biopsy was performed, whose macroscopy revealed a fibroelastic and multilobulated red-venous nodule. Histopathological analysis revealed cystic vascular structures with hemorrhagic content, with intraluminal papillary formations covered by an endothelium with soft cytology. No necrosis, cell pleomorphism, or mitotic figures were observed (Fig. 3). The histological appearance was compatible with IPEH (Masson's Tumor). In summary, IPEH or Masson's tumor is an infrequent benign intravascular lesion, caused by the proliferation of endothelial papillary structures organized into thrombi, constituting a reactive, traumatic, or undetermined cause in the context of venous stasis. Clinically, it resembles other types of subcutaneous tumors like angiomas, lipomas, angiolipomas, vascular malformations, cysts, and others. Histologically, its main differential diagnosis is with angiosarcoma, which is distinguished by its absence of necrosis, cellular atypia, and mitotic figures. Most of the case reports of this disease come from dentists, given the frequency of oral cavity involvement. Because of its benign nature, treatment depends on the location of the lesion and the symptoms it produces. In general, surgical resection is the treatment of choice, with good prognosis and low relapse rate. In research on this topic on the website of the Anais Brasileiros de Dermatologia, no previous publications were found in this journal, which was the reason for this report.

**Figure 1:**
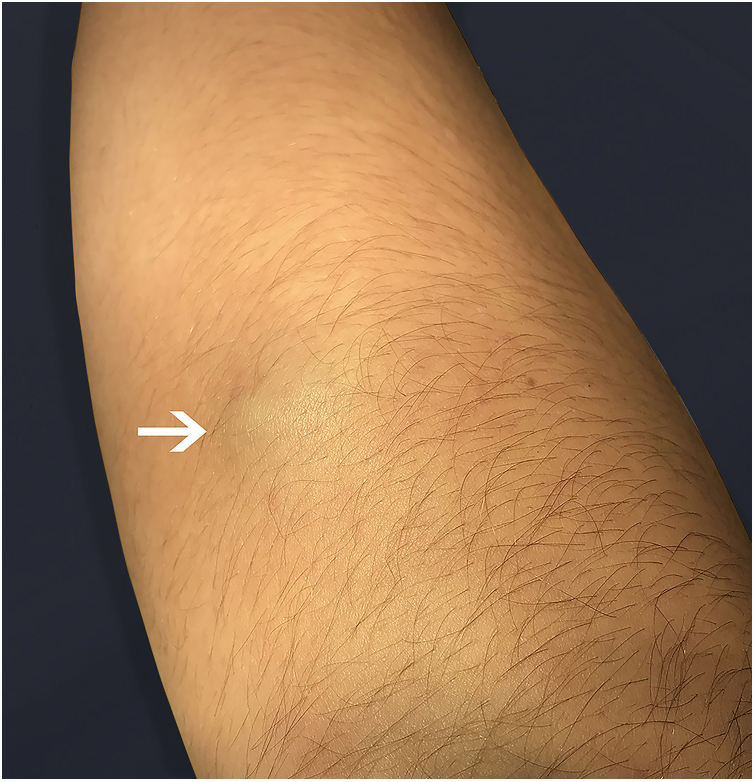
Fibroelastic subcutaneous nodule with imprecise limits and bluish coloration.

**Figure 2:**
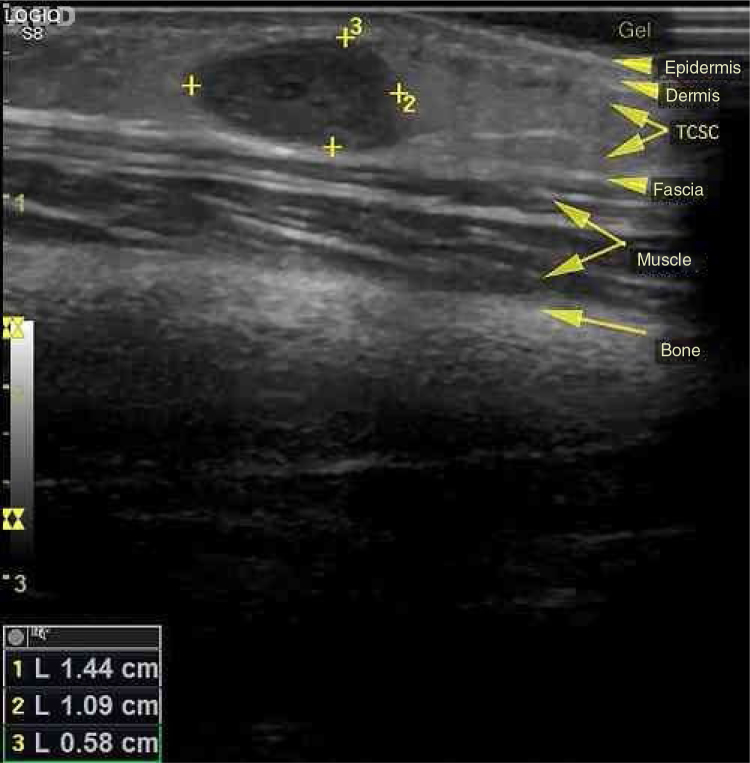
Hypoechoic subcutaneous nodule with precise limits.

**Figure 3:**
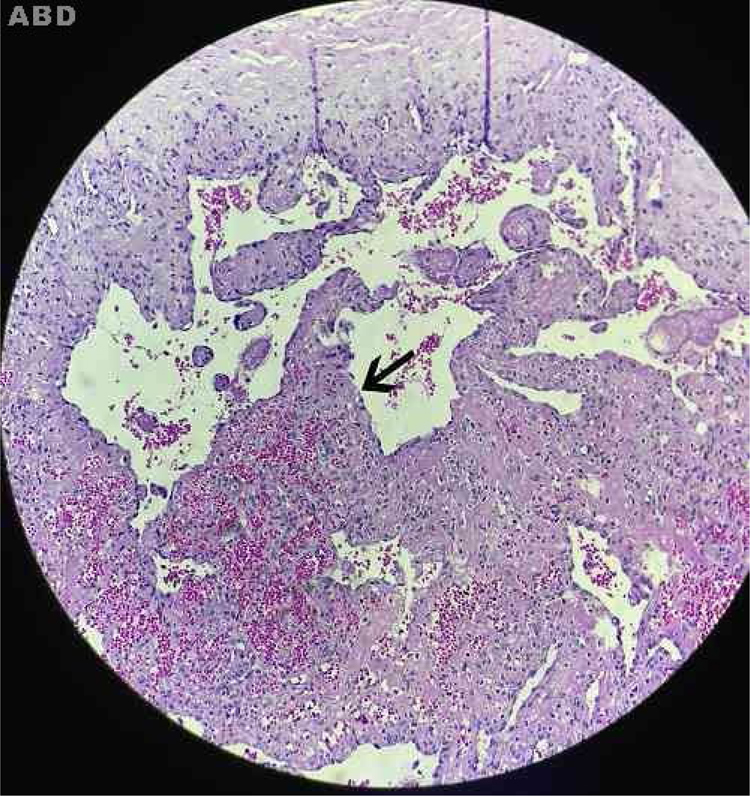
Cystic cavities lined by endothelium, presenting hemorrhagic content and papillary projections in the lumen (arrows). Absence of cellular atypia and necrosis (Hematoxylin & eosin, ×200).

## Financial Support

None declared.

## Author's contribution

*Sergio Emerson Sasso*: Approval of the final version of the manuscript; conception and planning of the study; elaboration and writing of the manuscript; obtaining, analyzing and interpreting the data; effective participation in research orientation; intellectual participation in propaedeutic and/or therapeutic conduct of the cases studied; critical review of the literature; critical review of the manuscript.

*Ana Paula Naspolini*: Approval of the final version of the manuscript; elaboration and writing of the manuscript; intellectual participation in propaedeutic and/or therapeutic conduct of the cases studied; critical review of the manuscript.

*Tassiana De Boit Milanez*: Obtaining, analyzing and interpreting the data.

*Guilherme Suchard*: Obtaining, analyzing and interpreting the data.

## Conflicts of interest

None declared.
